# Does the endotracheal tube insertion depth predicted by formulas in children have a good concordance with the ideal position observed by X-ray?

**DOI:** 10.5935/0103-507X.20200046

**Published:** 2020

**Authors:** Dayanna Letícia Silva Santos, Paulo Douglas de Oliveira Andrade, Evelim Leal de Freitas Dantas Gomes

**Affiliations:** 1 Faculdade de Fisioterapia e Terapia Ocupacional, Universidade Federal do Pará - Belém (PA), Brasil.; 2 Unidade de Terapia Intensiva Pediátrica, Fundação Hospital de Clínicas Gaspar Vianna - Belém (PA), Brasil.; 3 Universidade Nove de Julho - São Paulo (SP), Brasil.

**Keywords:** Intubation, intratracheal, Child, Infant, Thorax/imaging diagnosis, Intensive care units, pediatric, Intubação intratraqueal, Criança, Lactente, Tórax/diagnóstico por imagem, Unidades de terapia intensiva pediátrica

## Abstract

**Objective:**

To evaluate the effectiveness of the different formulas for estimating the insertion depth of an endotracheal tube in children.

**Methods:**

This was an observational and cross-sectional study that included children between 29 days and 2 years of age who were hospitalized in a pediatric intensive care unit and mechanically ventilated. The formulas based on height [(height/10) + 5], the inner diameter of the tube (endotracheal tube × 3), and weight (weight + 6) were evaluated to determine which of them showed better concordance with the ideal insertion depth of the endotracheal tube as evaluated by X-ray.

**Results:**

The correlation between the height-based calculation and the ideal depth observed on X-ray was strong, with r = 0.88, p < 0.05, and a concordance correlation coefficient of 0.88; the correlation between the weight-based calculation and depth on X-ray was r = 0.75, p < 0.05, and concordance correlation coefficient 0.43; and the correlation between endotracheal tube diameter-based calculation and depth on X-ray was r = 0.80, p < 0.05, and concordance correlation coefficient 0.78. Lin’s concordance correlation analysis indicated that the measurements showed weak concordance (< 0.90).

**Conclusion:**

The formulas that estimate the insertion depth of the endotracheal tube in children were not accurate and were discordant with the gold-standard method of X-ray evaluation. There is a need for a new method based on anthropometric variables (weight and height) and age that is effective in guiding health professionals of pediatric intensive care units at the time of intubation.

## Abstract (pt)

**Objetivo:**

Avaliar a efetividade dos diferentes métodos que estimam a profundidade de inserção do tubo orotraqueal em crianças.

**Métodos:**

Estudo observacional e transversal, no qual foram incluídas crianças entre 29 dias e 2 anos, internadas em uma unidade de terapia intensiva pediátrica e ventiladas mecanicamente. Foram avaliadas as fórmulas baseadas na altura [(altura/10) + 5], no diâmetro interno do tubo (tubo orotraqueal × 3) e peso (peso + 6), verificando-se qual delas apresentou melhor concordância com a profundidade ideal de inserção do tubo orotraqueal avaliado por radiografia.

**Resultados:**

A correlação entre a fórmula da altura e a profundidade ideal observada no raio X foi forte, com r = 0,88, p < 0,05 e o coeficiente de correlação de concordância de 0,88; a correlação do peso com a profundidade no raio X foi de r = 0,75, p < 0,05 e coeficiente de correlação de concordância de 0,43; e a fórmula do tubo orotraqueal × profundidade no raio X foi de r = 0,80, p < 0,05 e coeficiente de correlação de concordância de 0,78. A análise de concordância de Lin mostrou que as medidas apresentaram concordância fraca (< 0,90).

**Conclusão:**

As fórmulas que estimam a profundidade de inserção do tubo orotraqueal em crianças apresentaram-se pouco precisas e discordantes com o método padrão-ouro de avaliação pelo raio X, com necessidade da criação de um novo método, baseado nas variáveis antropométricas (peso e altura) e na idade, que seja eficaz para guiar os profissionais de saúde das unidades de terapia intensiva pediátricas, no momento da intubação.

## INTRODUCTION

The appropriate size and, especially, the insertion depth of an endotracheal tube (ET) should be accurately determined for pediatric patients because both deep and shallow intubation may result in complications.^(1)^

Several methods and formulas are indicated to calculate the ET insertion depth in children. The most commonly used formulas are based on the ET diameter, i.e., multiplying it by 3 (ET × 3); height, i.e., (height/10) + 5 (in cm); and weight, i.e., weight (in kg) + 6 (converting to cm).^(2-4)^

In children, the trachea is short, and the extension or flexion of the neck can cause ET displacement, with consequent accidental extubation or selective intubation. Ideally, the positioning of the tube in the middle of the trachea would allow a safety margin during movement of the head and neck. The tip of the ET should be positioned between the first thoracic vertebra (T1) and the carina.^(5,6)^

Bad positioning of the ET and wrong insertion depth are associated with several possible complications, including hypoxemia, atelectasis, selective intubation, barotrauma, pneumothorax, insufficient ventilation, vocal cord injury, air leak syndrome, accidental extubation, and even death. This inadequate positioning of the tube is a common occurrence in the neonatal and pediatric populations. Some authors report an incidence of 35-50% in patients younger than 1 year. In pediatric emergencies, approximately 30% of intubations are performed with inadequate placement of the tube. In pediatric intensive care units (ICU), this percentage is 13%. To confirm the correct position of the ET, chest X-ray remains the gold standard.^(1,7,8)^

The most common complications associated with tracheal intubation in the population below 2 years are selective tracheal intubation (31.1%), accidental extubation (25.6%), and hypoxia (41.1%).^(9-11)^ Atelectasis has also been documented as the most frequent complication, occurring in 36% of cases, with an incidence in 12% in children younger than 1 year old.^(9)^

Given the above, the identification of a formula that accurately estimates the ideal insertion depth of the ET at the time of intubation in pediatric patients becomes paramount for the prevention of these complications.^(9)^ Several formulas can be used, but there is no consensus on which one has better agreement with the best method.

The objective of this study was to assess which of the formulas most used in practice has greater concordance with the gold standard, X-ray positioning, in predicting the ideal insertion depth of the ET.

## METHODS

**Figure 1:**
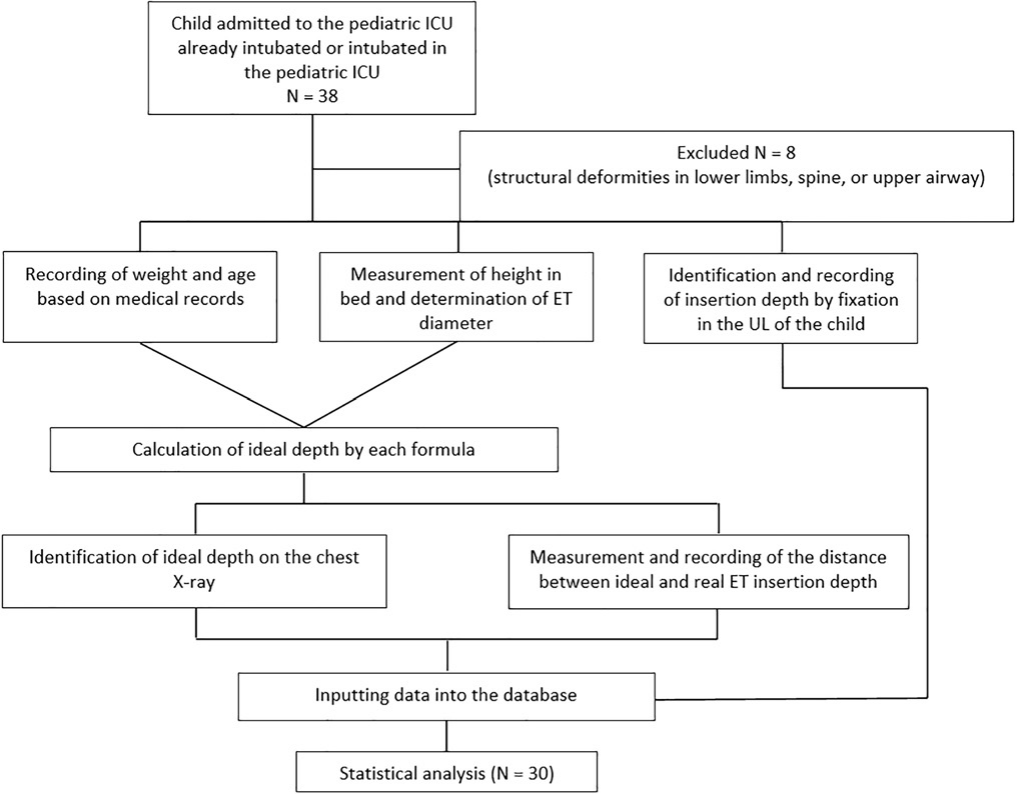
Flowchart. ICU - intensive care unit; ET - endotracheal tube; UL - upper lip.

A cross-sectional observational study was performed from September 2017 to January 2018 in a convenience sample of infants (29 days to 2 years) who were on mechanical ventilation with an ET in the pediatric ICU of the *Hospital das Clínicas Gaspar Vianna*, a cardiology hospital. This pediatric ICU had ten beds, one of which was an isolation bed, and was predominantly used by children with congenital heart diseases. Patients with deviation of the spine, airway (laryngeal, trachea, or bronchi) malformations, or lower limb deformities were excluded. The protocol was approved by the Research Ethics Committees of the *Instituto de Ciências da Saúde* of the *Universidade Federal do Pará*, under opinion number 2,248,923, and of the *Hospital das Clínicas Gaspar Vianna*, under opinion number 2,311,338. Figure 1 shows a flowchart with the sequence of evaluations.

The participants’ age, weight, height, and sex and the ET diameter and insertion depth were collected. Age, weight, and sex were obtained from the medical records. The child’s height was measured using a tape measure, measuring from the calcaneus to the tip of the skull in the dorsal decubitus position, with the bed completely horizontal.

The insertion depth of the ET was measured in centimeters, using the child’s upper lip as a reference. Later, the chest X-ray was evaluated on a X-ray viewer. For the X-ray to be considered adequate, the medial extremities of the clavicles needed to be equidistant from the spinal processes, and the carina had to be perfectly visible. The ideal depth was obtained by identifying the midpoint located between the first thoracic vertebra (T1) and the carina using a 30-cm ruler. The X-ray was also used to calculate the distance in centimeters between the ideal depth and the tip of the ET was. Measurement of the child’s height, identification of the insertion depth of the ET in the bed, and the determination of the ideal depth and the difference between real and ideal depths by chest X-ray were performed by the same professional.

For the purpose of our study, we defined, *a priori*, the mid-tracheal position as the space between the first thoracic (T1) and the carina.^(5)^

One table was input with these data, including the values obtained by calculating the three formulas analyzed: ET size (diameter in millimeters) × 3; child’s height (in centimeters)/10 + 5 and child’s weight (in kilograms) + 6 (converted to centimeters).

The analysis of these data aimed to establish the effectiveness of each formula based on the proximity of the value obtained to the ideal depth identified on X-ray.

### Statistical analysis and sample size calculation

Data analysis was performed using the Statistical Package for the Social Sciences (SPSS) version 23.0 and MedCalc. The data were stored in spreadsheets in Microsoft Excel® 2013, and each participant received a code to ensure their anonymity. The Shapiro-Wilk normality test was applied to analyze continuous variables. Those with a normal distribution were analyzed by Pearson’s correlation test. The following classification was used for the linear correlation analysis: r = 0, no correlation; r > 0 to 0.3, weak correlation; r > 0.3 to 0.6, moderate correlation; r > 0.6 to 0.9, strong correlation; r > 0.9 to 1, very strong correlation; r = 1, perfect correlation. After this correlation analysis, the significant variables were analyzed using a linear regression test to assess the size of the effect of the variables on ideal depth.

The categorical data were analyzed using Cochran’s Q test. A significant difference was accepted when p < 5% using Lin’s concordance correlation coefficient (CCC). For this analysis, the following categories were defined: CCC < 0.90, weak; 0.90 - 0.95, moderate; 0.95- 0.99, strong/substantial; and > 0.99 perfect. For the graphical analysis, the Bland-Altman plot was used. The sample size was calculated by analyzing the correlation between tracheal length and patient weight.^(11)^ This correlation was r = 0.82 (p < 0.05). Considering a two-tailed beta error of 0.1 and alpha error of 5%, with a power of 90%, two-tailed, the sample should comprise at least 15 children. We opted to collect a larger sample to account for potential losses. We used the weight for this calculation, rather than the height and age, because it had the weakest correlation, as those other variables had correlations with tracheal length of 0.9 and 0.86, respectively. In this way, the number of included individuals would also cover these outcomes.

## RESULTS

A total of 30 children aged 1 month to 2 years were admitted to the pediatric ICU of the *Hospital das Clínicas Gaspar Vianna*. All were diagnosed with congenital heart disease, and 16 (53.3%) were male. Their characteristics are shown in table 1. All chest X-rays were considered adequate based on the symmetry and visualization of the carina.

**Table 1:** Characteristics of the sample

Weight (kg)	5.37 ± 1.94
Height (cm)	62.9 ± 10.22
Age (months)	7.27 ± 6.44
Diagnoses	
Multiple heart defects	8 (26.6)
Tricuspid atresia	3 (10)
Ventricular septal defects	3 (10)
Coarctation of the aorta	2 (6.66)
PAPVR	1 (3.33)
AVSD	1 (3.33)
DORV	2 (6.66)
Pulmonary stenosis	2 (6.66)
Tetralogy of Fallot	1 (3.33)
Transposition of the great arteries	4 (13.33)
Single ventricle	1 (3.33)
Mitral valve regurgitation	1 (3.33)
Pulmonary hypertension	1 (3.33)

PAPVR - partial anomalous pulmonary venous return; AVSD - atrioventricular septal defect; DORV - double-outlet right ventricle. The results are expressed as the mean ± standard deviation or n (%).

**Figure 2:**
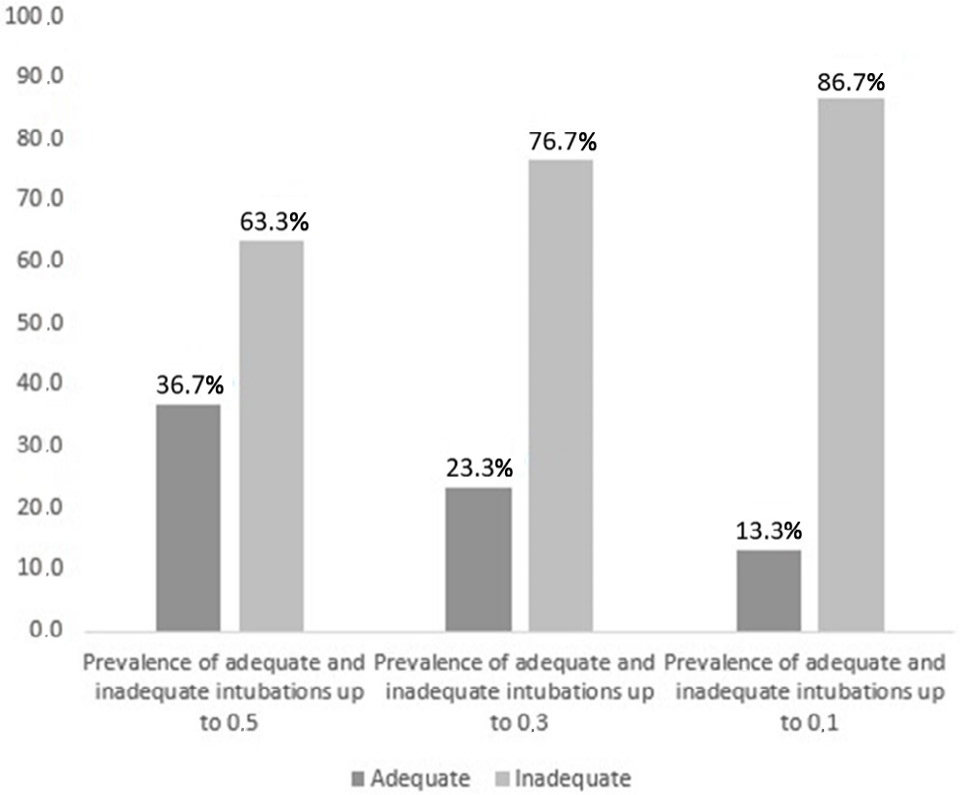
Prevalence of inadequate intubations.

The height formula was the one that was nearest the ideal point observed in the X-ray in 13/30 (43.3%) of the children, whereas this percentage was 11/30 (36.7%) for the ET formula and 9/30 (30%) for the weight formula. When allowing a difference of 0.1 cm relative to the ideal position for an intubation to be deemed adequate, it was found that 86.7% of the intubations were inadequate; when allowing a difference of 0.3 cm, 76.7% of the intubations were rated inadequate; and for a difference of 0.5 cm, 63.3% of the intubations were inadequate (Figure 2).

Cochran’s Q test showed no statistically significant difference between the formulas (p = 0.66), as they had similar proportions of times they were close to the ideal position for orotracheal intubation in pediatrics.

**Figure 3:**
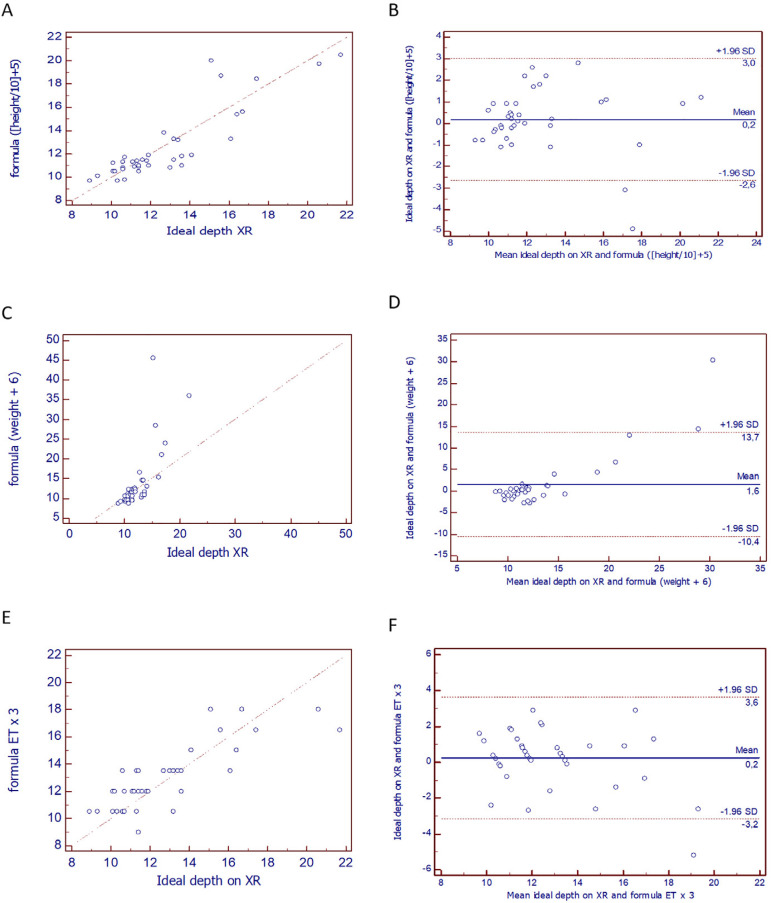
Correlation and agreement graphs: (A) Correlation of the height calculation with the X-ray depth: r = 0.89, p < 0.05; (B) Bland-Altman graph for the height calculation and the X-ray depth; (C) Correlation of the weight calculation with the X-ray depth: r = 0.75, p < 0.05; (D) Bland-Altman graph of the weight calculation and the X-ray depth; (E) Correlation of the endotracheal tube diameter calculation with the X-ray depth: r = 0.80, p < 0.05; (F) Bland-Altman graph for the endotracheal tube calculation and the X-ray depth.

The depth calculated by the height formula showed a strong correlation with the ideal depth (observed on X-ray), with r = 0.88, p < 0.05, and CCC = 0.88; the correlation between the weight-calculated depth and the depth on X-ray was also strong, with r = 0.75, p < 0.05, and CCC = 0.43; and the correlation between the ET formula calculation and the X-ray depth had r = 0.80, p < 0.05, and CCC = 0.78. Lin’s concordance analysis showed weak concordance (< 0.90) between the measurements. The height formula was the one closest to a moderate concordance. As for the Bland-Altman plots, Figure 3B, which was drawn using the height formula, shows a standard error of 0.2 and deviation of 2.6 to 3. This was the formula with the lowest standard error, but the deviation around this error was very large. Figure 3D, with the weight formula, shows that this measurement had the greatest error (1.6) and the smallest deviation (1.96). Figure 3F, of the measurement by the ET formula, shows a standard error of 0.2, but with deviation from 3.2 to 3.6. The three formulas showed moderate to strong correlations but were discordant by Bland-Altman analysis (Figure 3).

After the correlation analyses, linear regression was applied, and the possible relationships between the depth and the variables weight, height, and tube diameter were assessed. Weight (r = 0.69; p < 0.001; R^2^ = 0.47) showed a positive and highly significant correlation, with 47% of the variation in ideal depth being explained by weight. The height had r = 0.7, p < 0.001, and R^2^ = 0.49, which was also highly significant and explained 49% of the variation in ideal depth. The tube diameter showed a highly significant positive correlation, with r = 0.49, p = 0.003, and R^2^ = 0.24, and 24% of the variation in the ideal depth could be explained by the tube diameter. These calculations supported the other analyses in indicating that height was the measure that seemed to lead to the best positioning of the ET.

## DISCUSSION

The main finding of this study was that height was the variable that led to the most ideal calculation of ET depth, similar to the study that originated Morgan’s height formula,^(4)^ which observed that the length of the trachea increases linearly with height. However, in that study, the sample consisted of children older than 4 years of age, which explains the large percentage of mispositioned tubes in another study (> 25%)^(7)^ and our study (56.7%) from using the height formula, as these studies evaluated children under 4 years of age.

Another point to be highlighted is the inaccuracy of the weight and ET diameter formulas. The formulas showed lower CCCs than the height formula. Gill et al.^(12)^ confirmed this inaccuracy in the weight formula, especially in extremely low-birthweight infants. Additionally, a Cochrane systematic review from 2012^(13)^ already pointed to the insufficient evidence for these ideal-depth-prediction methods, with the X-ray still being the best method to position the ET.

This inaccuracy is confirmed by the high incidence of inadequate intubations, considering an error margin of 0.3cm and 0.5cm (76.7% and 63.3%, respectively). Thus, even with a margin of 0.5cm away from the ideal position, more than 60% of the intubations were still classified as inadequate. The greatest deviation was 3.6cm, which could lead to an immeasurable risk of complications for a trachea that is much smaller than the adult trachea.

A very important finding in the present study was the correlations between the insertion depth of the ET and the variables weight, height, and tube diameter. All variables had moderate to strong correlations, demonstrating the insufficiency of performing only the correlation analysis. Concordance analysis should also be performed; in this study, they ensured a more faithful reflection of the reality of the use of the formulas.

It is suggested, therefore, that a new method be devised for calculating the insertion depth of an ET in children that considers the child’s age along with weight and height. Only then will it be possible to optimize the accuracy of ET positioning at the time of intubation and prevent the numerous complications that can arise from this procedure in children.

A limitation of this study was the sample, which, although estimated by calculation, was still small. The results of other studies^(14-18)^ also indicate the inaccuracy of the formulas, meaning that our small sample size was probably not a bias that altered the results. Other limitations include the fact that the study was a single-center study, which may have influenced the anthropometric characteristics of the regional population; that the measurements were observer-dependent; and that there was no institutional protocol to determine the ideal ET size at the time of intubation, as Broselow pediatric emergency tape was not used in the evaluated ICU.

## CONCLUSION

The formulas that estimate the insertion depth of an endotracheal tube in children were not very accurate and deviated from the gold standard evaluation method (chest X-ray). It is necessary to create a new method based on anthropometric variables (weight and height) and age that can reliably guide health professionals in pediatric intensive care units and surgical centers at the time of intubation.
